# A Unique Presentation of Birt-Hogg-Dube Syndrome

**DOI:** 10.7759/cureus.17227

**Published:** 2021-08-16

**Authors:** Emily Medhus, Michael Siegel, Joseph Boscia

**Affiliations:** 1 Pediatrics, Edward Via College of Osteopathic Medicine-Carolinas, Spartanburg, USA; 2 Internal Medicine, Edward Via College of Osteopathic Medicine-Carolinas, Spartanburg, USA; 3 Pulmonary and Critical Care Medicine, Edward Via College of Osteopathic Medicine-Carolinas, Spartanburg, USA

**Keywords:** birt-hogg-dubé syndrome, flcn, mtor, pneumothorax (ptx), fibrofolliculomas, carcinomas renal cell, sebaceous cyst, genodermatosis, perifollicular fibroma

## Abstract

Birt-Hogg-Dube (BHD) syndrome is a rare autosomal dominant condition identified by the triad of cutaneous fibrofolliculomas, pulmonary cysts, and renal cell carcinoma. The vast majority of patients with BHD syndrome initially present with spontaneous pneumothorax. This unique case describes a patient with BHD syndrome who presented with sebaceous cysts and perifollicular fibromas. The evaluation by dermatology is what led to his diagnosis. Expanding the clinical presentation of BHD syndrome to encompass a variety of skin findings could help with recognizing these patients before they suffer the serious complications of renal carcinoma and pneumothorax.

## Introduction

Birt-Hogg-Dube (BHD) syndrome is an autosomal dominant condition associated with multisystem effects [1]. Clinical manifestations include cutaneous fibrofolliculomas, pulmonary cysts, spontaneous pneumothorax, and renal cell carcinoma. The majority of patients initially present with spontaneous pneumothorax and usually have a significant family history of the disease, however, that is not always the case [2]. 

A genetic mutation of the Folliculin (FLCN) gene has been identified and linked to BHD syndrome. It typically occurs as a germline mutation, but spontaneous mutations have also been reported. The FLCN gene codes for a tumor suppressor protein, Folliculin, which acts as a regulator of the mTOR pathway of cell growth [3]. This is a rare genetic condition affecting about 600 families worldwide [4]. Diagnosing a patient with BHD can be challenging considering the limited exposure to cases in everyday practice combined with a variety of clinical manifestations that could present. Here, we describe the unusual presentation and associated comorbidities of a patient with BHD with no known prior family history of the disease or symptoms of the disease. As in this case, it often takes a multidisciplinary approach to diagnose a rare genetic condition, such as BHD syndrome.

## Case presentation

A 52-year-old Caucasian male presented to dermatology for initial evaluation of multiple recurring cysts, nodules, and papules. For several years, his family medicine physician treated him for sebaceous cysts (by local excision) located mostly on his upper back, but recurrence and complexity warranted further evaluation. At this visit, he described the presence of cysts that would come and go, occasionally becoming inflamed and tender. He also inquired about numerous white facial papules that were otherwise asymptomatic.

This patient had struggled with inflammatory cysts and other skin lesions for many years before being tested for BHD. In 2011, he had a large 11 cm x 7 cm painful fluctuant cystic structure located over the upper thoracic spine, along with about 20 small scattered furuncles distributed across his back. The examination also revealed a chronic non-inflamed sebaceous cyst, 2 cm x 4 cm, located anterior to the left knee. In 2015, the patient developed another sebaceous cyst, 1 cm in size, located near his right jaw. The smaller nodules scattered across his back were still present and would come and go.

Physical exam findings from the dermatology visit showed a 1 cm firm mobile nodule on the right cheek, a 2.5 cm firm mobile nodule on the right upper back (epidermoid cyst), and several 1 cm firm mobile nodules on the right superior back. Additionally, the patient had numerous white 0.2-0.4 cm papules distributed across his nose, cheeks, and forehead.

The numerous white facial papules raised concern for genodermatosis, specifically BHD syndrome or Cowden syndrome. Interestingly, the patient reported they had been present for many years, but they were not mentioned in previous records. A shave biopsy of one of the papules was sent for histopathological analysis and identified as a perifollicular fibroma. This piece of information was instrumental in leading toward an eventual diagnosis. The further genetic evaluation confirmed the presence of an FLCN gene mutation and the diagnosis of BHD syndrome was finally made.

**Figure 1:**
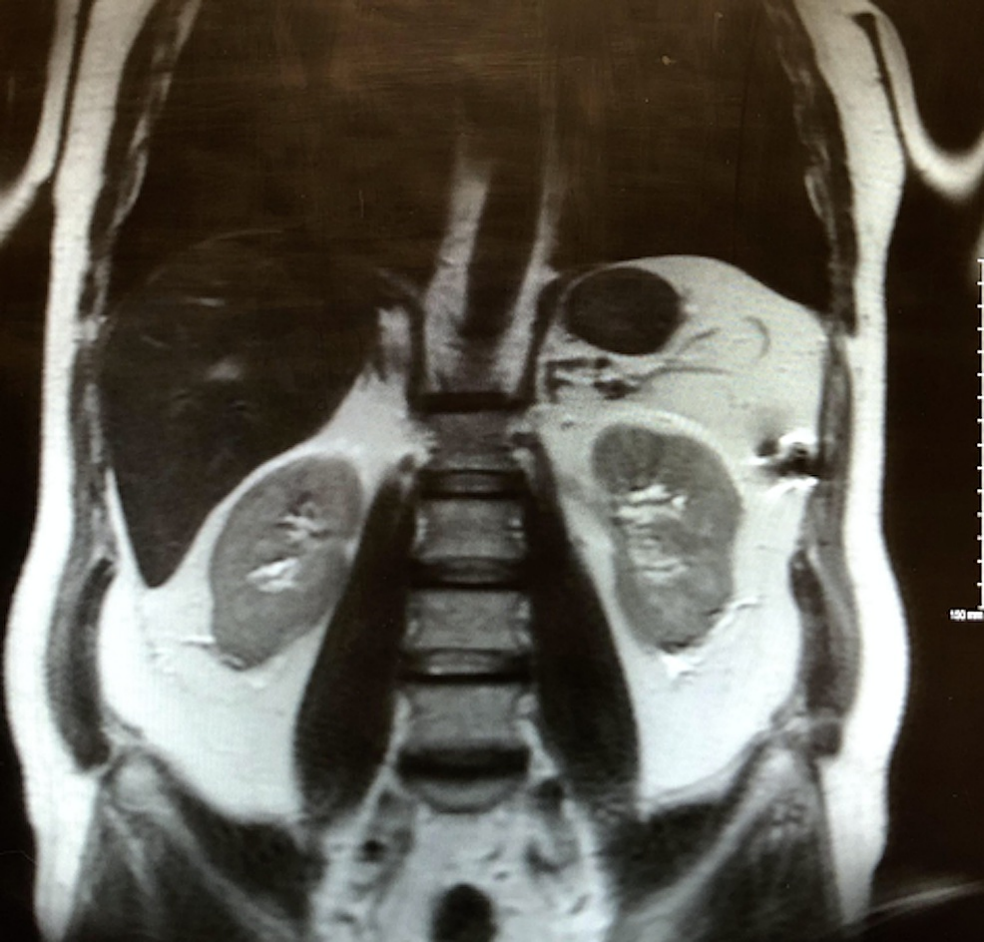
MRI abdomen showing no evidence for renal cell carcinoma

Further testing was performed to determine if other risk factors such as pneumothorax or renal cell carcinoma were present. The patient was counseled on symptoms of pneumothorax. Figure 1 shows an MRI of the abdomen of the patient, which showed no masses suggesting renal cell carcinoma.

## Discussion

This case describes an atypical presentation of BHD syndrome with unique skin features and a lack of initial pneumothorax. There were many challenges in making the diagnosis of BHD syndrome in this case. The patient lacked significant family history and did not present with spontaneous pneumothorax, typical features of BHD [2]. Additionally, his primary skin concerns focused on sebaceous cysts and inflammatory lesions, not the classic fibrofolliculomas. This might explain why it took several years before he was identified and diagnosed.

The wide variety of clinical manifestations and presentations of BHD syndrome makes detecting it challenging. While studies show that a majority of patients present with pneumothorax, it has also been reported that over 85% of BHD patients have classic skin fibrofolliculomas [3]. Is it possible that patients with BHD-associated complications could have been identified earlier by examining their skin? If they had been identified earlier, is it possible that they could have prepared for or minimized the risk of pneumothorax by quitting smoking? Recognizing the cutaneous features associated with BHD prior to the onset of complications can improve the prognosis of these patients. Therefore, having a high clinical index of suspicion in patients with numerous white facial papules is of utmost importance.

Sebaceous cysts are an extremely rare cutaneous manifestation in the presentation of BHD syndrome [5]. It is unclear whether or not his cystic skin lesions represent a variant form of BHD or an isolated finding. Further research identifying other patients with similar cystic manifestations should be done in order to make that association. If an association is made, that may provide a novel approach to identifying these patients, leading to earlier diagnosis and prevention of serious comorbidities.

## Conclusions

This is a rare presentation for BHD syndrome. The occurrence of inflammatory cysts along with fibrofolliculomas presents a novel occurrence in BHD syndrome. The determination of fibrofolliculomas as the presenting symptoms allows the patient to be vigilant of the expected symptoms from BHD syndrome. The strength of this report is in the interdisciplinary approach to patient care. Evaluations by physicians of many different specialties provided a full picture of the patient’s journey with BHD. It is this interdisciplinary approach that allows both a refined management strategy for the patient and a comprehensive case study.

## References

[1] Aivaz O, Berkman S, Middelton L (2015). Comedonal and cystic fibrofolliculomas in Birt-Hogg-Dube syndrome. JAMA Dermatol.

[2] Zbar B, Alvord WG, Glenn G (2002). Risk of renal and colonic neoplasms and spontaneous pneumothorax in the Birt-Hogg-Dubé syndrome. Cancer Epidemiol Biomarkers Prev.

[3] Schmidt LS, Linehan WM (2015). Molecular genetics and clinical features of Birt-Hogg-Dubé syndrome. Nat Rev Urol.

[4] Jensen DK, Villumsen A, Skytte AB, Madsen MG, Sommerlund M, Bendstrup E (2017). Birt-Hogg-Dubé syndrome: a case report and a review of the literature. Eur Clin Respir J.

[5] Kluger N, Giraud S, Coupier I (2010). Birt-Hogg-Dubé syndrome: clinical and genetic studies of 10 French families. Br J Dermatol.

